# Can allele-specific loop-mediated isothermal amplification be used for rapid detection of target-site herbicide resistance in *Lolium* spp.?

**DOI:** 10.1186/s13007-023-00989-0

**Published:** 2023-02-07

**Authors:** Silvia Panozzo, Silvia Farinati, Maurizio Sattin, Laura Scarabel

**Affiliations:** 1 Institute for Sustainable Plant Protection (IPSP) - National Research Council (CNR), viale dell’Università 16, 35020 Legnaro, PD Italy; 2 Department of Agronomy, Food, Natural Resources, Animals and Environment (DAFNAE), University of Padova, Padova, Italy

**Keywords:** LAMP, ALS inhibitors, ACCase inhibitors, Herbicide resistance, Target-site resistance detection, Ryegrass

## Abstract

**Background:**

Herbicide resistance is one of the threats to modern agriculture and its early detection is one of the most effective components for sustainable resistance management strategies. Many techniques have been used for target-site-resistance detection. Allele-Specific Loop-Mediated Isothermal Amplification (AS-LAMP) was evaluated as a possible rapid diagnostic method for acetyl-CoA carboxylase (ACCase) and acetolactate synthase (ALS) inhibiting herbicides resistance in *Lolium* spp.

**Results:**

AS-LAMP protocols were set up for the most frequent mutations responsible for herbicide resistance to ALS (positions 197, 376 and 574) and ACCase (positions 1781, 2041 and 2078) inhibitors in previously characterized and genotyped *Lolium* spp. populations. A validation step on new putative resistant populations gave the overview of a possible use of this tool for herbicide resistance diagnosis in *Lolium* spp. Regarding the ACCase inhibitor pinoxaden, in more than 65% of the analysed plants, the LAMP assay and genotyping were in keeping, whereas the results were not consistent when ALS inhibitors resistance was considered. Limitations on the use of this technique for herbicide resistance detection in the allogamous *Lolium* spp. are discussed.

**Conclusions:**

The LAMP method used for the detection of target-site resistance in weed species could be applicable with target genes that do not have high genetic variability, such as *ACCase* gene in *Lolium* spp.

**Supplementary Information:**

The online version contains supplementary material available at 10.1186/s13007-023-00989-0.

## Background

Herbicide resistance affects many different cropping systems worldwide and can determine a high yield reduction [1]. Therefore, there is a need to rapidly detect the presence of resistant weeds to prevent further resistance selection and avoid the use of herbicides that are no longer effective. This in turn will facilitate the adoption of adequate weed control strategies.

Herbicide resistance can be due to mutation(s) in the herbicide target gene conferring amino-acid changes in the protein and consequent inhibition of herbicide binding, the so-called target-site herbicide resistance (TSR) [2]. The development of molecular detection tools able to recognize the mutations identified in the weed populations is of great interest and over the years a range of techniques were developed [3, 4]. These techniques include PCR–RFLP (Restriction Fragment Length Polymorphism) and PASA (PCR Amplification of Specific Alleles) [5], CAPS (Cleaved Amplified Polymorphic Sequence) and dCAPS (derived CAPS) [6, 7], Real-Time PCR [8] and pyrosequencing [9]. Overall, the major drawback of these methods is that they are time consuming, require expensive chemicals, special thermal cyclers and are not suitable for rapid resistance detection in the field.

**Fig. 1:**
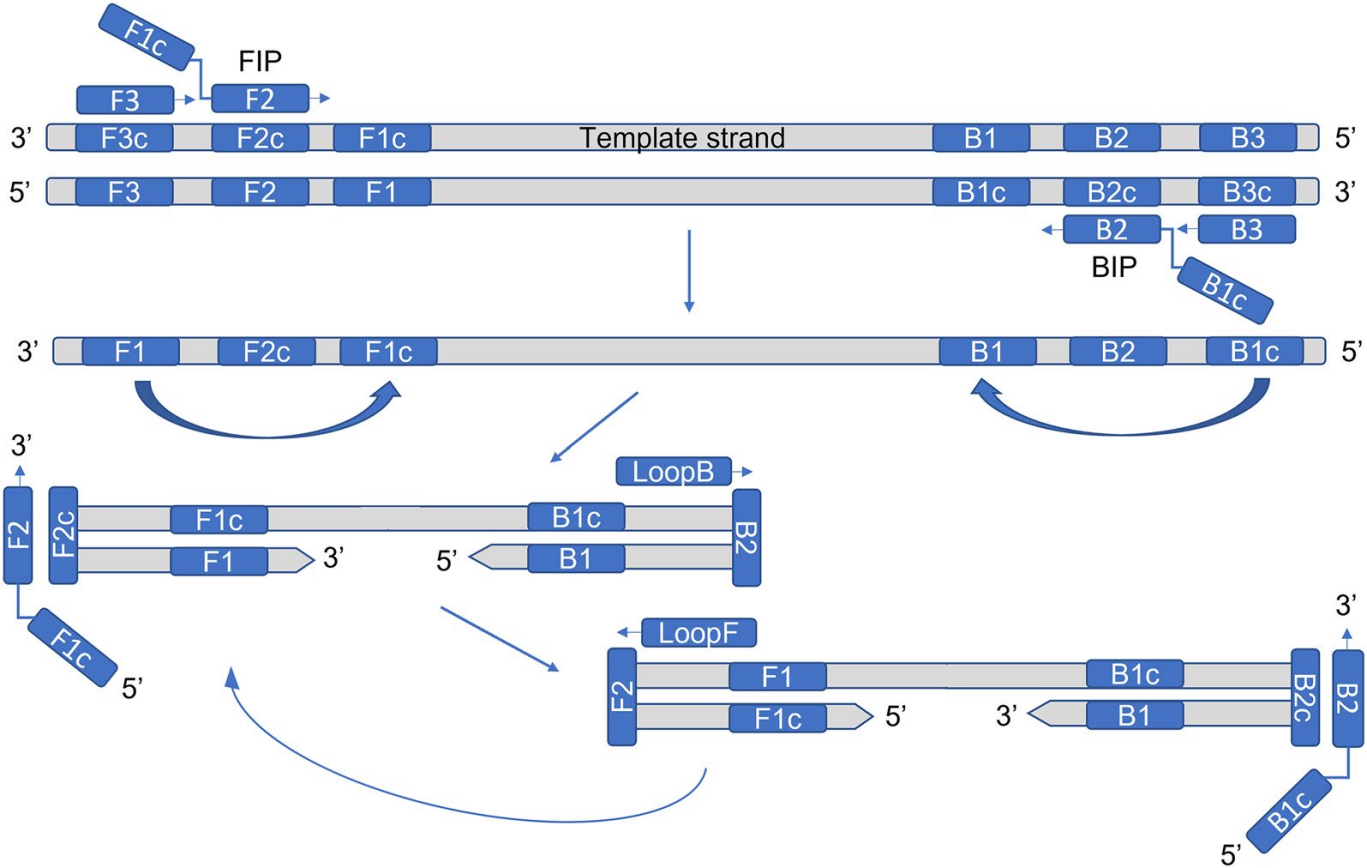
Diagram of LAMP method and primers involved. Four species-specific primers are involved in the template strand amplification: two external primers, F3 (forward outer primer) and B3 (backward outer primer), and two internal primers, FIP (forward inner primer) and BIP (backward inner primer) -which are made-up linking two complementary regions of the template strand- that during the amplification step will create a loop which is exponentially amplified. Other two primers, LoopB and LoopF, may be designed for stabilization of the amplification

A relatively novel molecular method is the Loop-Mediated Isothermal Amplification (LAMP) assay, which rapidly amplifies target nucleic acids under isothermal conditions (i.e. a single incubation temperature) [10]. In contrast to the PCR techniques, the LAMP method is more specific because it uses 4 to 6 different primers that specifically recognize 6 to 8 discrete regions of the target gene (template strand) (Fig. 1) [11]. Both endpoint detection and real-time monitoring of the LAMP reaction can be done by various approaches. Since its introduction, the LAMP technique has been applied for detecting point mutations conferring resistance mainly to pathogens, like fungicide resistance [12, 13]. Despite the agronomic issue of herbicide resistant weeds increasing, to date, the LAMP method has been applied only once for the detection of five point mutations in *Beckmannia syzigachne* resistant to fenoxaprop-p-ethyl [14, 15].

Ryegrass species (*Lolium* spp.) are common weeds infesting many crops including winter cereals. *Lolium rigidum* Gaud. (Rigid ryegrass) and *Lolium multiflorum* Lam. (Italian ryegrass) are the two most common species of *Lolium* and are troublesome weeds in cereal crops as well as in orchards, olive groves and vineyards. Because *Lolium* species are often mixed in the field, not always easily identifiable, and respond similarly to herbicides [16], in many cases a population is defined as “*Lolium* spp.”. This is also emphasized by its biological self-incompatibility reproduction system, which concurs to a high level of genetic variability [17].

In winter cereal crops, control of *Lolium* spp. largely depends on ALS (acetolactate synthase) and ACCase (acetyl-CoA carboxylase) inhibiting herbicides. The repeated use of these herbicides has led to the evolution of *Lolium* spp. resistant cases. To date, multiple resistance to ALS and ACCase inhibitors have been reported in many countries worldwide [18]. This represents a major practical agronomic problem because it significantly reduces the herbicides available for *Lolium* spp. control. Several resistance mechanisms have been identified in *Lolium* spp., and among them target-site resistance (TSR) and detoxification of the herbicide (i.e. enhanced metabolic resistance) are the main ones [19, 20]. Many of the known herbicide resistance-endowing mutations in the *ALS* and *ACCase* genes were found in *Lolium* spp. Regarding *ALS* gene, mutations in three positions of the gene were documented: five allelic variations in position Pro-197 (Pro to Ala, Arg, Gln, Leu or Ser) [21], Asp-376-Glu [22] and Trp-574-Leu [19]. Resistance to ACCase inhibitors mainly results from mutations at seven *ACCase* codon positions: Ile-1781-Leu, Trp-1999-Cys, Trp-2027-Cys, two allelic variations in position 2041 (Ile to Asn or Val), Asp-2078-Gly, Cys-2088-Arg and Gly-2096-Ala [23–27]. Herbicide resistance surveys conducted at large scale revealed that ACCase-resistant *Lolium* spp. populations from the United Kingdom and Australia had a predominance of Asp-2078-Gly and Ile-2041-Asn mutations respectively [28, 29]. Instead, an Italian study conducted on five ACCase-resistant *Lolium* populations showed that the mutation Ile-1781-Leu was predominant [25].

The aim of this research was to develop a LAMP protocol able to detect the most common mutations (hereafter called “target mutations”) endowing resistance to *ACCase* (Ile-1781-Leu, Ile-2041-Asn and Asp-2078-Gly) and *ALS* (Pro-197-Ser, Asp-376-Glu and Trp-574-Leu) inhibitors in *Lolium* spp. populations and evaluate its real effectiveness as a rapid diagnostic method for herbicide resistance.

## Methods

### Plant material

Twelve populations of *Lolium* spp. collected in cereals fields in Italy, Denmark and Greece with different patterns and levels of resistance were considered for the LAMP set-up. These populations were chosen because previous studies revealed that they had a high frequency of resistant plants due to an altered herbicide target gene, and different allelic variants correlated to the resistance phenotypes were identified [30]. The most frequent mutations endowing ACCase inhibitors resistance were reported at positions 1781, 2041 and 2078 of the *ACCase* gene and the most frequent mutations endowing ALS inhibitors resistance were reported at positions 197, 376 and 574 of the *ALS* gene.

### Allele-specific loop-mediated isothermal amplification (AS-LAMP) assay set-up

#### Primer sets design and testing

In our Allele-Specific (AS) LAMP method, two sets of LAMP primers (identified as wild type–WT—and mutated–MUT) are provided for distinguishing between two different nucleotides in a specific point of the sequence of a target gene [31]. The WT/MUT primer sets were designed using as input sequences generated during the genotyping of the twelve characterized *Lolium* spp. populations briefly described in “Plant material” section and reported in Table 1 as ‘reference tested plants’. The sequences used are reported in a specific repository [32], whereas the Additional file 1 reports the details of the chromatograms of the 'reference tested plants’ in the mutation points considered for the *ALS* and *ACCase* genes. The following tests, to define quality and specificity of each primer set, were performed using the same known WT and MUT on homo/heterozygous genotypes previously characterized by Sanger DNA sequencing method and used for the design of the primers.

**Table 1:** LAMP primer sets designed for each target mutation with indication of the strategy used

Gene	Target mutation	Primer sets	Reference tested plants
*ALS*	197	#1_FIP5′	IT595.4 (−/−) GR20.7 (+ / +)
		#2_BIP5′	
	376	#1_FIP5′	IT533.4 (−/−) IT595.1 (+ / +)
		#2_FIP3′	
	574	#1_FIP3′	IT595.1 (−/−) DK47.6 (+ / +)
		#2_BIP3′	
		#3_FIP5′	
		#4_BIP5′	
*ACCase*	1781	#1_FIP5′	IT620.10 (−/−) GR9.6 (+ / +)
		#2_BIP5′	
	2041	#1_FIP5′	IT620.10 (−/−) GR20.10 (+ / +)
		#2_BIP5′	
	2078	#1_FIP5′	IT620.10 (−/−) GR20.6 ( ±)
		#2_BIP5′	

Reference plants used for LAMP primer sets design, as well as tested during protocol set-up phase, and the genotype of target mutations (−/− for WT, + / + for MUT homozygous and ± for MUT heterozygous) are reported for each plant (see Additional file 1 for the chromatograms of the 'Reference tested plants’ in the different target mutations considered for *ALS* and *ACCase* gene, respectively)

For each target mutation two sets of specific primers were designed, one primer set specific for the nucleotide which identifies the wild type (WT, responsible for the susceptible, S, phenotype) and one primer set specific for the nucleotide which identifies the mutated (MUT, responsible for the resistant, R, phenotype). Four-six species-specific LAMP oligo primers (see Fig. 1) for *Lolium* spp. (external primers F3 and B3, internal primers FIP and BIP, and, optionally, loop primers F-Loop and B-Loop) were designed using Primer explorer V5 software (http://primerexplorer.jp/lampv5e/index.html). While F3 and B3 primers are common for a target mutation, the FIP and BIP primers have been modified to give specificity for the WT or MUT allele, respectively. Several strategies were used: in the primers FIP and BIP the mutation was included in the 5′ end of F1c or B1c (FIP5′ and BIP5′ primers, respectively) or in the 3′ end of F2 or B2 (FIP3′ and BIP3′ primers, respectively) (Fig. 1); sometimes, to enhance the specificity of the primer, an additional mismatch was deliberately introduced at the third position from the 3′ end of the primer [33].

The complete list of WT/MUT primer sets designed for the different target mutations are reported in Additional file 2.

#### LAMP reaction setting

Real-time LAMP assays were conducted on a Genie II instrument (OptiGene, Horsham, United Kingdom) in 25 µl reaction mixtures containing 15 µl of isothermal master mix at a 1X concentration (OptiGene), 200 nM each external primer, 2 µM each internal primer and 1 µM each loop primer. The isothermal master mix contained a fluorescent double-stranded DNA binding dye for the real-time detection of the results.

To evaluate the sensitivity of the LAMP assays, serial dilutions of template DNA were tested, and assays were optimized also in terms of reaction time and temperature. After amplification, the nature of the amplification products was confirmed by subjecting the reactions to a slow annealing step (0.05 °C per s) from 95 °C to 75 °C with fluorescence monitoring.

### AS-LAMP assay validation

#### Selection of ALS and ACCase resistant plants

For the LAMP validation step, seven additional *Lolium* spp. populations collected in Italian wheat fields (codes 576, 594, 602, 648, 651, 670 and 678), as well as a susceptible check (code 204L), were considered. Seeds were collected from at least 30 plants per field, cleaned and stored in double paper envelopes in the dark at 4 °C. Populations were firstly characterized for herbicide resistance following the protocol reported in Panozzo et al. [34]. Briefly, seeds of each population were pre-germinated, transplanted into pots in the greenhouse, and seedlings at 3–4 leaf stage were sprayed with two herbicides belonging to the two different Sites of Action (SoA) [35] at the field dose (1X) to determine the resistance pattern: the ACCase inhibitor pinoxaden (1X = 45 g a.i. ha^−1^) and one mixture of two ALS inhibitors mesosulfuron-methyl + iodosulfuron-methyl sodium (1X = 15 + 3 g a.i. ha^−1^). Resistance status was evaluated as percentage of plant survival and VEB (Visual Estimated Biomass), in relation to the untreated check, 4 weeks after treatment.

#### AS-LAMP assay and subsequent genotyping

From 5 to 10 resistant plants of each population were sampled and used both for LAMP analyses and the following genotyping for the detection of *ALS* and *ACCase* point mutations. Genomic DNA (gDNA) was extracted from 100 mg of young leaf tissue (one leaf per plant) using the CTAB (cetyltrimethylammonium bromide) method [36] from plants that had survived the herbicide treatments. Nucleic acid concentration was measured using a NanoDrop 2000c Spectrophotometer (NanoDrop Products, USA).

Discriminating LAMP primer sets (identified in “Primer sets design and testing” section) for detecting *ACCase* mutations 1781, 2041 and 2078 were tested on gDNA extracted from plants surviving pinoxaden treatment, whereas discriminating LAMP primer sets for detecting *ALS* mutations 197, 376 and 574 were tested on gDNA extracted from plants that survived mesosulfuron + iodosulfuron treatment.

To confirm the results of the LAMP assay, i.e. presence of mutation(s) endowing herbicide resistance, amplifications of *ALS* and *ACCase* genes were obtained with PCR reactions using GoTaq®G2 Hot Start Polymerase (Promega, USA) following the manufacturer’s instructions. Details of the genotyping procedure are described in Scarabel et al. [30]. The primer pairs used to amplify the full *ALS* gene sequence were LOL_ALS_F (5′-CCGCAAGGGCGCCGACATCCTCGT-3′) and LOL_ALS_R (5′-CGAAATCCTGCCATCACCTTCCAT-3′), whereas the primer pairs used to amplify the CT domain of the *ACCase* gene sequence were acclr9 (5′-ATGGTAGCCTGGATCTTGGACATG-3′) and acclr6 (5′-GGAAGTGTCATGCAATTCAGCAA-3′) [37]. Amplicons were sequenced by BMR Genomics (Padova, Italy) using primers LOL_ALS_F and ALS_LOL_FS (5′-TCCATCACCAAGCACAACTACCTC-3′) for *ALS* gene, and LOL_FOR (5′- CTGTCTGAAGAAGACTATGGCCG-3′) and LOL_FOR_SEQ (5′- GAGGTGGCTCAGCTATGTTCCTG-3′) for *ACCase* gene, respectively.

## Results and discussion

As preliminary test, it was decided to apply the AS-LAMP to previously genotyped *Lolium* spp. populations. Generally, the most frequent mutations were reported in positions 1781 (47% of the mutated plants found), 2041 (20%) and 2078 (10%) of the *ACCase* gene (the remaining 23% of the mutated plants found included other known mutations, i.e. in positions 2027, 2088 and 2096) and in positions 197 (43%), 376 (15%) and 574 (36%) of the *ALS* gene (the remaining 6% of the mutated plants included other known mutations, i.e. in positions 122 and 205) [30].

### AS-LAMP assay set-up

The testing phase revealed that the best operating (amplification) condition was obtained with 25 ng of template gDNA added per reaction and with the reaction held at 60 °C for 60 min.

Using the different strategies, Primers Explorer V5 software allowed more than one pair of LAMP primer sets to be designed (one for the WT and one for the MUT) around each target mutation in *ALS* and *ACCase* gene sequences (Table 1 and Additional file 2). It should be noted that when more than one allelic variant was found, the most frequent mutated allele for that position was considered: e.g. position 2041 of *ACCase* gene (WT = ATT, Ile) had two mutant allelic variants known, AAT (Asn) and GTT (Val), but primer sets for that position were designed to be specific only to detect the most frequent triplet AAT; position 197 of *ALS* gene had several allelic variants known, only in *Lolium* populations included in this study five different allelic variants were detected, but primer sets were designed to specifically recognize the most frequent variant CCG/TCG (Pro/Ser) (detected in 26% of the plants mutated in position 197).

**Fig. 2:**
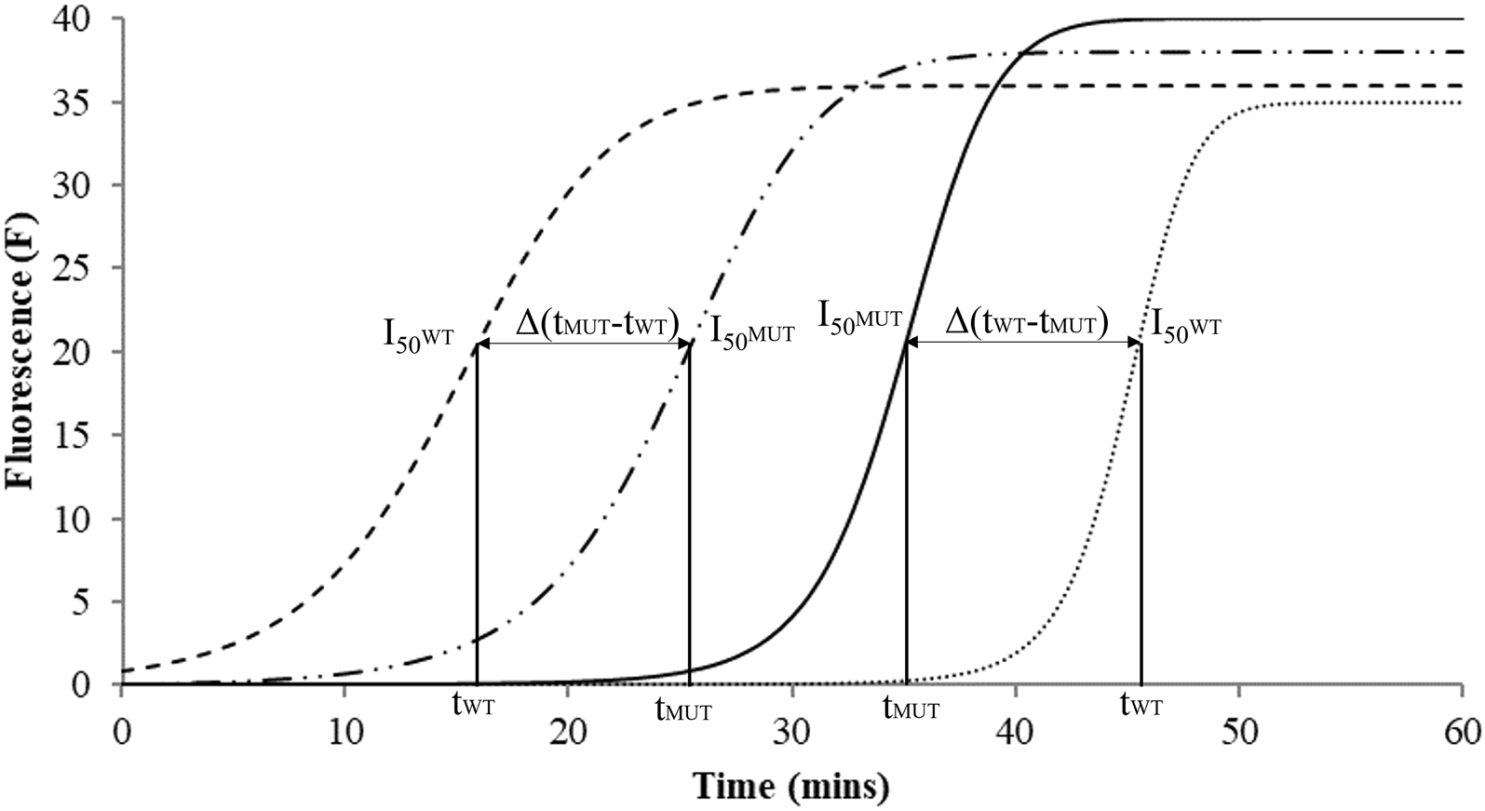
Scheme of amplification plots obtained after specific LAMP reaction in the GeneII instrument. Amplification plots represent the reaction trend of fluorescence emitted during the time (run of 60 min) for a sample with a WT genotype tested with WT set primers (dashed line) and MUT set primers (dashed line with dots). When a Δ(t_MUT_-t_WT_) of at least 5 min was observed, the sample was ascribed as WT. The two curves on the right represent the opposite situation: a MUT genotype tested with MUT set primers (continuous line) and WT set primers (dotted lines), in this case the Δt is in favour of the MUT primer set

As the test was based on allele specific sequences, it was expected to obtain an on/off response, i.e. amplification for WT allele with primer set specific for WT allele but no amplification for MUT allele with the same primer set (and vice versa). Instead, in all tested samples only a delay was obtained in the beginning of the exponential phase of the curve when, for example, a WT genotype was tested with MUT primers and vice versa (Fig. 2). The delay time (i.e. evaluated on the inflection point, I_50_, of the amplification curve), was observed when a WT sample was questioned with MUT primer set in comparison to the relative WT primer set (and vice versa). After several tests on known genotypes, it was agreed to choose a delay time of at least 5 min (Δt) of target amplification as the main parameter to discriminate the working success of a specific primer set. In addition, the technical factors typically linked to success of an amplification reaction, as the presence of a single melting curve for each primer set investigated and a negative control (i.e. with the addition of water rather than unknown genomic sample), helped in this phase of interpretation of the results. In the Additional file 2 the operating conditions of each primer set are reported; only sets producing a single melting curve and Δt > 5 min were considered for following analyses. Regarding the *ALS* gene, the set with strategy FIP5’ (i.e. specificity of the primer FIP was put at the 5′ end of primer F1c) for position 197 and 376 and the set with strategy BIP5’ (i.e. specificity of the primer BIP was put at the 5′ end of primer B1c) for position 574 were the most successful sets for distinguishing the allelic variants. For the *ACCase* gene, the set with the strategy BIP5’ resulted the best for all tested nucleotide substitutions.

### Selection of ALS and ACCase resistant plants

Once the best working primer sets had been identified for each target mutation, they were tested on gDNA extracted from plants which showed a resistant phenotype to ACCase or ALS inhibitors (as described in “Selection of ALS and ACCase resistant plants” section) but for which the resistance mechanism is unknown.

**Fig. 3:**
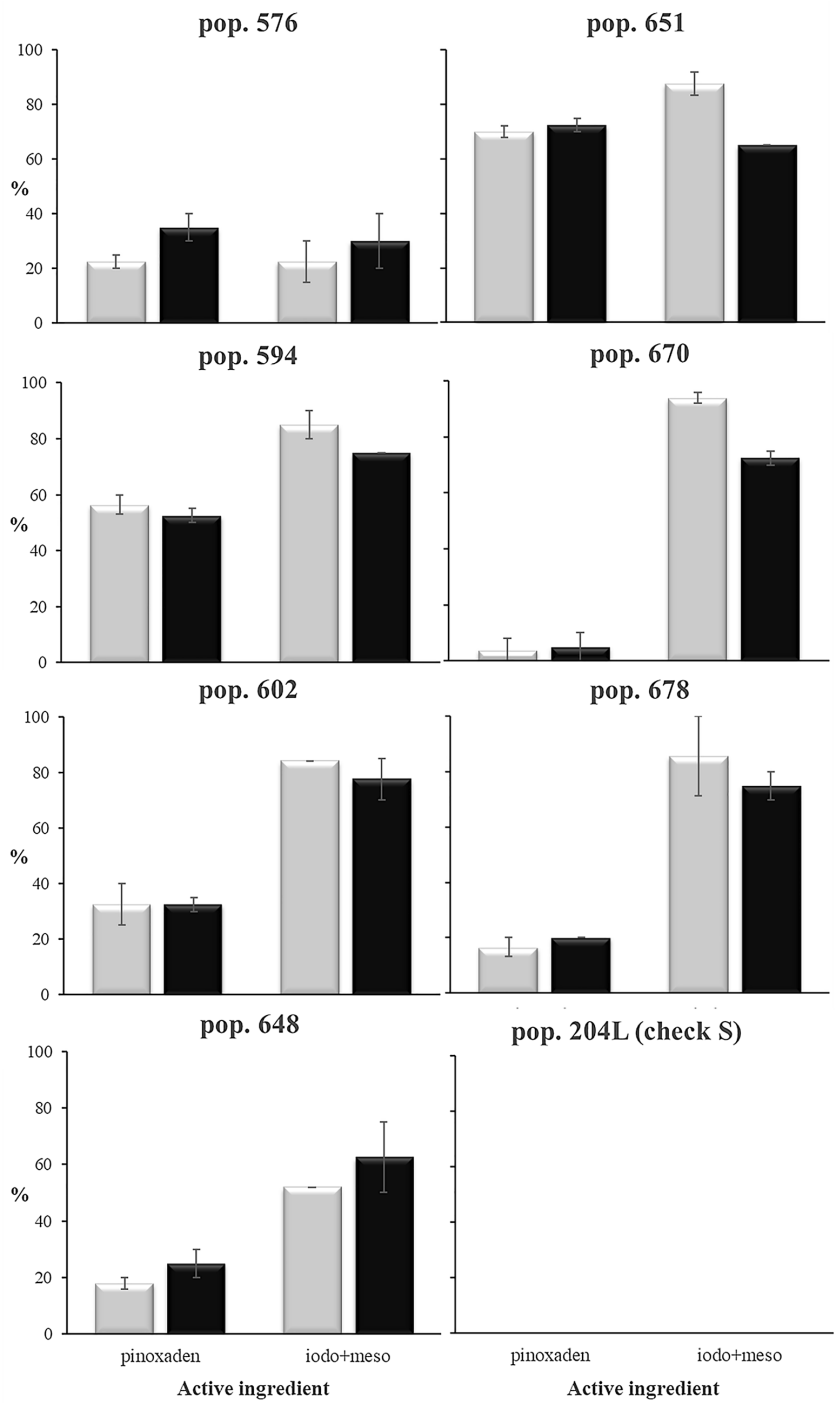
Results of the greenhouse bioassay to determine the resistance pattern of the seven populations included in the AS-LAMP validation step. Percentage of plant survival (grey bars) and Visual Estimated Biomass (VEB) (black bars) of populations treated with pinoxaden (ACCase inhibitor) and iodosulfuron + mesosulfuron (ALS inhibitors) at the reccomended field dose (1x). Vertical thin bars represent the standard error

The bioassay performed on the populations included in the AS-LAMP validation step showed that the susceptible check 204L was completely controlled by both herbicides. All test populations, except population 576, had a survival rate to iodosulfuron + mesosulfuron of between 52 and 94%. Two populations, 670 and 678, were highly resistant to ALS inhibitors with more than 85% of plants surviving but were still adequately controlled by ACCase inhibitors (Fig. 3), whereas the other five populations tested (651, 576, 594, 602 and 648) were multi-resistant to the ALS inhibitors iodosulfuron + mesosulfuron and to the ACCase inhibitor pinoxaden (Fig. 3). Some pictures showing the plants which survived the different herbicides, as well as those of the susceptible check 204L, were reported in Additional file 3.

### LAMP validation and genotyping

The AS-LAMP assays on the three most frequent point mutations in *ALS* locus (positions 197, 376 and 574) were performed on 5 plants of ALS resistant populations 670 and 678 and the multi-resistant population 651 (Fig. 3). The investigations on the three most frequent point mutations in *ACCase* locus (positions 1781, 2041 and 2078) were instead conducted on 10 plants derived from population 651 which was highly resistant to pinoxaden and on 5 plants from populations 576, 594, 602 and 648 which showed different level of resistance to the same herbicide. This was because the different level of resistance may be due to different point mutations in the *ACCase* gene.

**Fig. 4:**
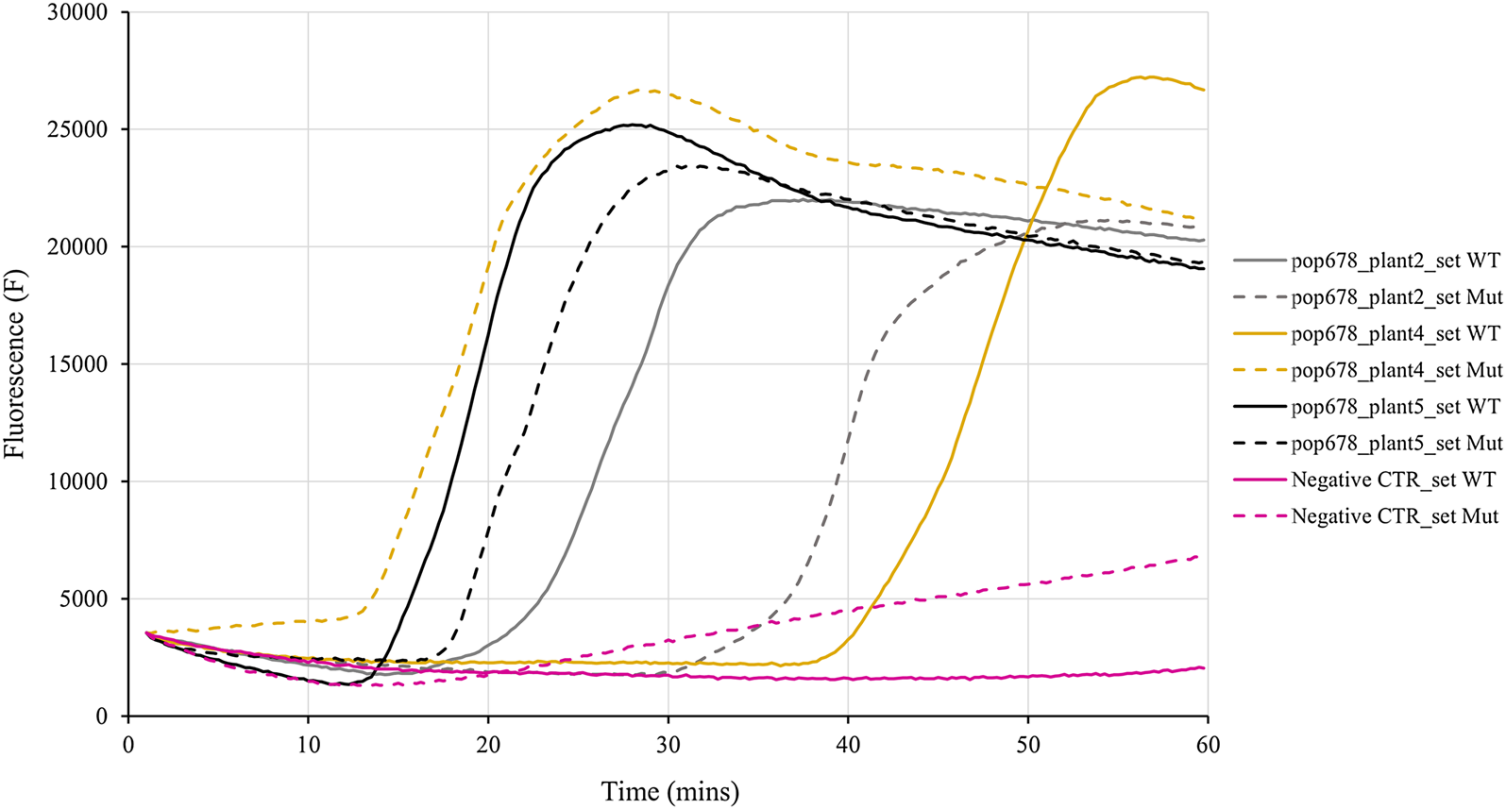
Example of amplification plots of AS-LAMP reaction on point mutation 376 of *ALS* gene. Three plants of populations 678 (plants 2, 4 and 5) were analysed with the specific WT primer set (continuous lines) and MUT primer set (dashed lines) for the detection of mutation 376. A negative control (i.e. only water was included in the reaction mix) for both primer sets was also included (pink lines). To facilitate the reading, the lines were coloured in grey when the prediction was correct and therefore consistent with the genotyping, in yellow when they didn’t and in black when the results were not determined (Δt < 5 min)

**Fig. 5:**
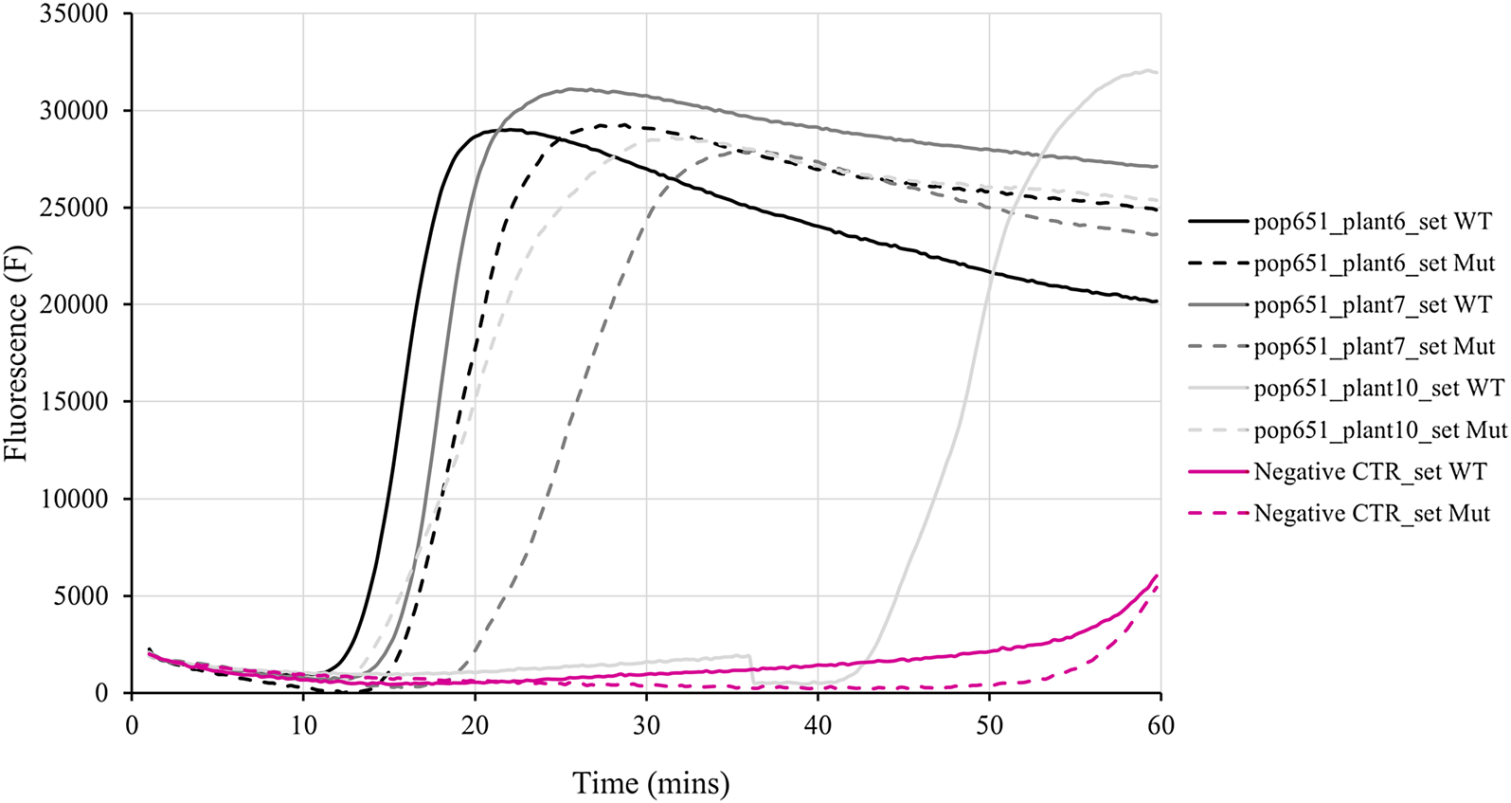
Example of amplification plots of AS-LAMP reaction on point mutation 2041 of *ACCase* gene. Three plants of populations 651 (plants 6, 7 and 10) were analysed with the specific WT primer set (continuous lines) and MUT primer set (dashed lines) for the detection of mutation 2041. A negative control (i.e. only water was included in the reaction mix) for both primer sets was also included (pink lines). To facilitate the reading, the lines were coloured in grey when the prediction was correct and therefore consistent with the genotyping and in black when the results were not determined (Δt < 5 min)

The AS-LAMP assays were performed and analysed based on discriminative criteria found on ‘delay of amplification’ threshold, the results obtained are summarized in Table 2. To be as conservative as possible, the result “nd” has been introduced to indicate that it was impossible to distinguish the WT from MUT allele using the ‘delay of amplification’ and that the parameter of Δt > 5 between the curves was not respected (i.e. results were not clear or ambiguous). Two examples of amplification plots reporting results of AS-LAMP for the analyses of point mutation 376 of *ALS* gene in population 678 and point mutation 2041 of *ACCase* gene in population 651 are reported in Figs. 4 and 5, respectively. In Fig. 4 it is observable that plant 2 was a WT (Δt > 5 min with amplification with WT set primer which started before the amplification with MUT set primer), plant 4 was a MUT plant (Δt > 5 min with amplification with MUT set primer which started before the amplification with WT set primer) and the ambiguity of result for plant 5 (Δt < 5 and therefore impossibility to classify the sample as WT or MUT). In Fig. 5 it is observable that plant 7 was a WT, plant 10 a MUT and for plant 6 the result was indeterminate.

**Table 2:** Comparison of results obtained from LAMP reactions and genotyping for identifying A) *ALS* and B) *ACCase* allelic variants in resistant *Lolium* spp. plants

Locus	Target mutation	Method	A) Population and plant numbers														
			651					670					678				
			1	2	3	4	5	1	2	3	4	5	1	2	3	4	5
*ALS*	197	LAMP	nd	nd	**wt**	nd	**wt**	nd	nd	nd	nd	nd	nd	**wt**	nd	**mut**	*mut*
		Genotyping	–	C(C/A)G	**CAG**	–	**CAG**	–	–	–	–	–	–	**C(C/A)G**	–	–	*CAG*
	376	LAMP	*wt*	/	/	*wt*	*wt*	*wt*	*wt*	*wt*	nd	nd	*wt*	*wt*	*wt*	**mut**	nd
		Genotyping	–	–	–	–	–	–	–	–	–	–	–	–	–	–	–
	574	LAMP	nd	nd	nd	*wt*	*wt*	nd	nd	nd	*wt*	*wt*	*wt*	*wt*	*wt*	*wt*	*wt*
		Genotyping	–	–	–	–	–	–	–	–	–	–	–	–	–	–	–

Locus	Target mutation	Method	B) Population and plant numbers														
			651										576				
			1	2	3	4	5	6	7	8	9	10	1	2	3	4	5
*ACCase*	1781	LAMP	nd	*mut*	*wt*	/	*mut*	*mut*	*mut*	*wt*	*mut*	*wt*	/	*mut*	nd	*wt*	*mut*
		Genotyping	(A/T)TA	*(A/T)TA*	–	/	*TTA*	*(A/T)TA*	*(A/T)TA*	–	*(A/T)TA*	–	TTA	*(A/T)TA*	(A/C)TA	-	*(A/T)TA*
	2041	LAMP	*wt*	nd	*mut*	/	*wt*	nd	*wt*	**wt**	*wt*	*mut*	*wt*	*wt*	nd	*wt*	*wt*
		Genotyping	–	A(T/A)T	*A(T/A)T*	/	–	A(T/G)T	–	**A(T/A)T**	–	*AAT*	–	–	A(T/A)T	–	–
	2078	LAMP	nd	*wt*	nd	/	nd	*wt*	*wt*	*mut*	*wt*	*wt*	*wt*	/	nd	*wt*	**mut**
		Genotyping	–	–	G(A/G)T	/	–	–	–	*G(A/G)T*	–	–	–	–	–	–	–

			**594**					**602**					**648**				
			**1**	**2**	**3**	**4**	**5**	**1**	**2**	**3**	**4**	**5**	**2**	**3**	**4**	**5**	**6**
*ACCase*	1781	LAMP	*mut*	*mut*	**wt**	**wt**	*mut*	*wt*	*wt*	*wt*	**mut**	/	/	/	*wt*	nd	*wt*
		Genotyping	*TTA*	*TTA*	**TTA**	**(A/T)TA**	*(A/T)TA*	–	–	–	–	–	–	–	–	–	–
	2041	LAMP	*wt*	nd	nd	*wt*	*wt*	*wt*	*wt*	*wt*	nd	*wt*	nd	**mut**	*wt*	*wt*	*wt*
		Genotyping	–	–	–	–	–	–	–	–	–	–	–	–	–	–	–
	2078	LAMP	*wt*	*wt*	*wt*	*wt*	*wt*	*wt*	*wt*	*wt*	*wt*	**mut**	*wt*	*wt*	nd	nd	*mut*
		Genotyping	–	–	–	–	–	–	–	–	–	–	–	–	–	–	*G(A/G)T*

LAMP results are distinguished in: *wt*  wild type allele and *mut*  mutated allele; *nd*  impossible to distinguish the WT from MUT allele (i.e. results were not clear or ambiguous); *slashes (/)*  no amplification curves were obtained with both sets of primers

Genotyping results reported a dash (−) when the genotype had the WT sequence into the specific codon and the mutated triplet was indicated when a substitution into the WT triplet was detected in the sequence; slashes (/) indicates that sequence was not readable. To facilitate the reading, in italics when the results were consistent between genotyping and LAMP assays, in bold when the two investigation methods gave contrasting results

The results from the LAMP assays were compared with those obtained with the genotyping of *ALS* gene (Table 2A) and *ACCase* gene (Table 2B) on the same plants.

Overall, when considering the *ALS* gene, only 19 out of 45 data (in italics) showed consistency between genotyping and LAMP analyses (Table 2A). For many plants the LAMP results were not clear enough to assert if a plant had a specific mutation or not (nd) indicating that the requirement of the Δt of at least 5 min was not met. For the target mutation 197, the results were consistent in only one sample out of 15 tested (i.e. plant 5 of population 678) and in four cases the results were in contrast (point mutation present in the sequence of the gene but WT for LAMP, e.g. plant 651–3, or vice versa, e.g. plant 678–4). None of the plants of populations 651, 670 and 678 carried point mutations 376 and 574 (Table 2A); LAMP results confirmed the WT genotype only for 9 plants out of 15 analysed for each point mutation. This result could be explained by imputing a strategic role to the nucleotide context: *ALS* gene has sequences rich in GC [38] which often makes primers design difficult or leads to obtaining poor quality primers.

A better consistency between LAMP and genotyping data was detected for the *ACCase* gene (Table 2B). A total of 29 plants were genotyped (plant 651–4 sequence was not readable): for target mutation 1781, results were consistent in 19 plants out of 29, whereas in 3 plants results were in contrast and in 3 plants results of LAMP were not clear. Unfortunately, reaction did not start in 4 plants. For position 2041, results were consistent in 20 plants out of 29, whereas in 7 plants results of LAMP were not clear and only in 2 plants were results in contrast; for position 2078, results were consistent in 20 plants out of 29, whereas in 6 plants LAMP results were confusing, in 2 plants LAMP and genotyping results were in contrast, and in 1 case reaction did not start. It is noteworthy that in 2 plants of population 602 (602–1 and 602–5) different mutations were found (in position 2088 and 2027 of the *ACCase* gene, respectively) with respect to those detected with LAMP method.

Overall, in more than 65% of cases LAMP and genotyping results were consistent, while in 7–10% of the cases they were not. Of course, while the percentage of plants in which the reaction did not start is never very high (3–14%), the cases in which it is not possible to give an answer with the LAMP method reaches even 24% of the target mutations 2041 (Table 3).

**Table 3:** Overall comparison between LAMP and genotyping results for the *ACCase* gene

LAMP and genotyping results		*ACCase* position		
		1781	2041	2078
Consistent	n. plants	19	20	20
	%	66	69	69
In contrast	n. plants	3	2	2
	%	10	7	7
LAMP results were not clear	n. plants	3	7	6
	%	10	24	21
LAMP reaction did not start	n. plants	4	–	1
	%	14	–	3
Total	n. plant	29	29	29

Number of plants and percentage where results were consistent/in contrast, or LAMP gave results not clear, or it did not give results

### Limitations of LAMP method in herbicide resistance detection

The main limitation of LAMP method in the genotyping of TSR mutations in *Lolium* spp. is that it is feasible only to detect point mutations where a unique allelic variant was reported: the specific primer (FIP or BIP) is designed to detect only a specific allelic variant, whereas plants including a different allelic variant in the same point will be recognised as not mutated (e.g. position 2041 of *ACCase* gene has two mutant allelic variant known as AAT and GTT, but primer BIP5’ was designed only to detect the triplet AAT). In fact, the result of the only plant carrying the triplet GTT was not correct with the LAMP method (plant 651–6, Table 2B). The same is true for position 197 of the *ALS* gene where 5 different allelic variants were detected in this study, and many others are reported in the literature [18, 30]. On the other hand, we detected some false positives (plants responded as mutated in the LAMP method which did not carry the mutation, e.g. the above-mentioned plant 602–5), though in low percentage (7–10%).

Another variable that needs to be considered is primer specificity: the primer sets WT and MUT are not always specific enough to give a clear distinction between the I_50_ of the two curves in the amplification plot (Fig. 2). In *Lolium* spp. this is most likely linked to the biological characteristics of the species: their genetic self-incompatibility means that they are obligate allogamous and the different species can interbreed (i.e. *Lolium rigidum* and *Lolium multiflorum*) leading to highly variable nucleotide contexts [17]. Proof of this is also the large number of point mutations that may underlie resistance to different herbicides SoA in these species [39]. This is also a possible explanation for why we never observed an on/off response and needed to put a threshold for the Δt of the curves to assign a sample to WT or MUT genotype. When this requisite was not applied, two almost overlapping curves were obtained, and the results could not be assigned (“nd” in Table 2). The only other published case of LAMP use for the genotyping of TSR in a weed is in *Beckmannia syzigachne,* which is a self-pollinating species [15]. The same was recently confirmed also for other self-pollinating diploid species, *Amaranthus* spp. [40]. Another attempt to use LAMP method to detect the ALS mutations in *Echinochloa phyllopogon* by Pan et al. [15] was a failure, possibly due to *E. phyllopogon* biology (i.e. it is an allotetraploid grass species with multiple copies of the *ALS* genes).

## Conclusions

The AS-LAMP method was designed to detect three *ACCase* mutations responsible for resistance in *Lolium* spp. but was not accurate enough to be successfully applied at large scale in the field. The development of the LAMP protocol to detect three *ALS* mutations responsible for resistance failed. Nonetheless, LAMP remains a potentially powerful method, but only in certain conditions. The biology of the species involved, and the genetic variability of the target genes are fundamental aspects for setting up a LAMP protocol. Contrary to what is reported in the literature for insects, plant diseases and a diploid autogamous weed, in our study the methodology showed several limitations. The LAMP assay of an allogamous weed species with high genetic variability such as *Lolium* spp. appears hardly applicable and limited to genes which are intrinsically less variable (i.e., *ACCase* gene).

## Supplementary Information

**Additional file 1. **Graphic representations of allelic variants of each target mutation: chromatogram details of the different ‘Reference tested plants’ focused on the mutation points of the *ALS* and *ACCase* genes.

**Additional file 2. **Completed list of the primers used in AS-LAMP set-up: the different primer sets designed for each target mutation are reported.

**Additional file 3.** Visual results of herbicides screening tests performed on some Italian Lolium spp. populations collected in wheat fields.

## Data Availability

Data will be made available on acceptance of this manuscript at the Institute for Sustainable Plant Protection (IPSP), research unit of Legnaro (PD), from the corresponding author on reasonable request. Part of the data analysed during the current study are available in the Mendeley repository “gDNA chromatograms of reference plants used for the development of AS-LAMP in *Lolium* spp. resistant to herbicides”, https://doi.org/10.17632/34rx4md6k6.1.

## Abbreviations

AS-LAMP: Allele-specific loop-mediated isothermal amplification
ALS: Acetolactate synthase
ACCase: Acetyl-CoA carboxylase
TSR: Target-site resistance
F3: Forward outer primer
B3: Backward outer primer
FIP: Forward inner primer
BIP: Backward inner primer
WT: Wild type
MUT: Mutated
S: Susceptible
R: Resistant
SoA: Site of Action
VEB: Visual Estimated Biomass
gDNA: genomic DNA
CTAB: Cetyltrimethylammonium bromide
