# Supplementary material for: Can allele-specific loop-mediated isothermal amplification be used for rapid detection of target-site herbicide resistance in Lolium spp.?
Source: Plant Methods. 2023 Feb 7;19:14. doi: 10.1186/s13007-023-00989-0 (PMC9906911; doi:10.1186/s13007-023-00989-0)
Supplement: Supplementary file 1 — Additional file 1. Graphic representations of allelic variants of each target mutation: chromatogram details of the different ‘Reference tested plants’ focused on the mutation points of the ALS and ACCase genes. [file 13007_2023_989_MOESM1_ESM.docx]

**Graphic representations of allelic variants of each target mutation considered in the AS-LAMP set-up**

Chromatogram details of the different ‘Reference tested plants’ (see Table 1 in the main text) focused on the mutation points of the ALS gene (up) and ACCase gene (down). The triplets encoding for the amino acids which are involved in herbicide resistance are highlighted in blue. The wt (wild type) sequences are reported on the left, whereas the mut (mutated) sequences are reported on the right. The complete sequences of these samples were used for the design of the AS-LAMP WT/MUT primer sets for each target mutation and the corresponding gDNAs for the following tests to define quality and specificity of each primer set. The code of each chromatogram identifies: the origin of the sample (IT = Italy, GR = Greece, DK = Denmark), the population and plant number (e.g. 595.4, 20.7, etc.), status of the sample (wt or mut) and number of target mutation (e.g. 197, 376, etc.).
