# Supplementary material for: Can allele-specific loop-mediated isothermal amplification be used for rapid detection of target-site herbicide resistance in Lolium spp.?
Source: Plant Methods. 2023 Feb 7;19:14. doi: 10.1186/s13007-023-00989-0 (PMC9906911; doi:10.1186/s13007-023-00989-0)

**Visual results of herbicides screening tests performed on some Italian *Lolium* spp. populations collected in wheat fields**

In each picture, pots with plants of different Lolium spp populations are included. In each picture: front, pots of the susceptible check 204L; back, pots of a test population (670, 678 and 651, respectively). A text label identify the treatment for each pot: NT = not treated control; pinoxaden 1x = treatment with 45 g a.i. ha^-1^; iodo+meso 1x = treatment with mesosulfuron-methyl 15 g a.i. ha^-1^ + iodosulfuron-methyl 3 g a.i. ha^-1^. Pictures were taken 28 days after treatment.

**Population 670**: controlled by pinoxaden (ACCase inhibitor) , but highly resistant to iodo+meso (ALS inhibitors), see also Fig. 3 in the main text


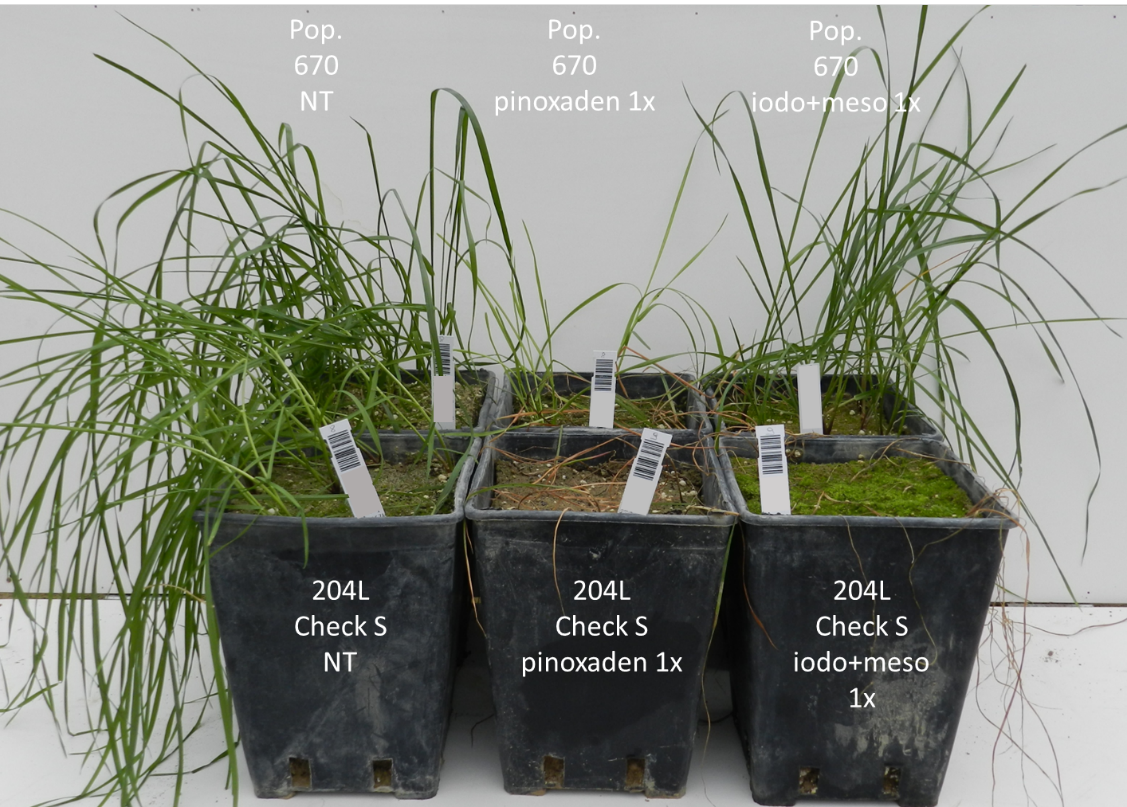


**Population 678**: controlled by pinoxaden (ACCase inhibitor) , but highly resistant to iodo+meso (ALS inhibitors), see also Fig. 3 in the main text


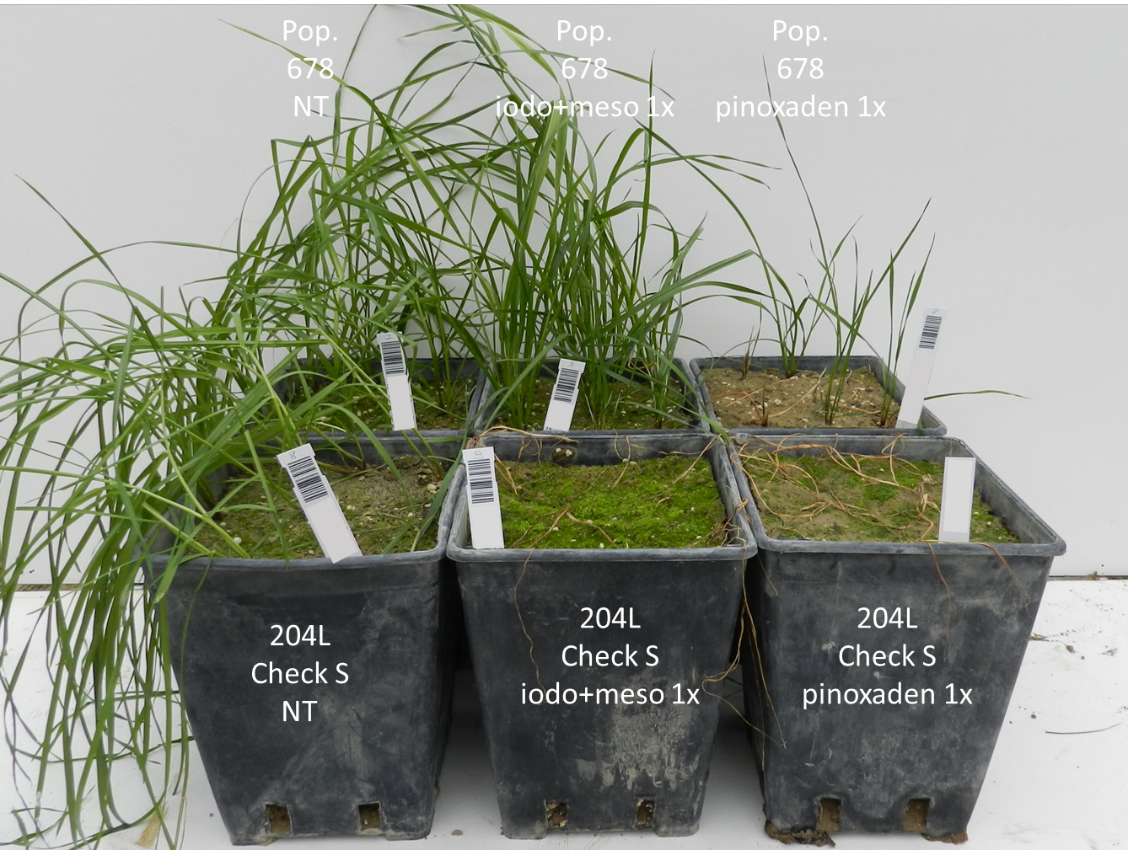


**Population 651**: highly resistant to both pinoxaden (ACCase inhibitor) and iodo+meso (ALS inhibitors), see also Fig. 3 in the main text


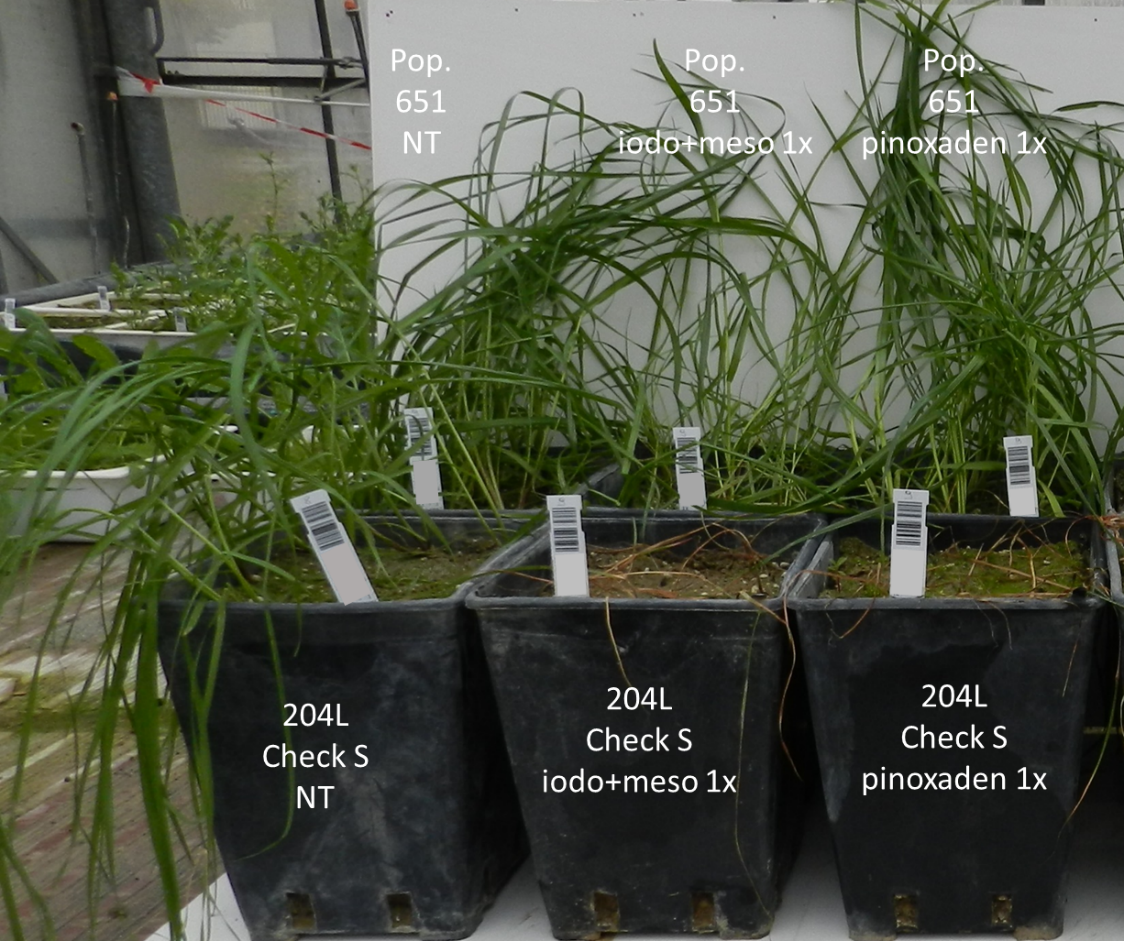

Supplement: Supplementary file 3 — Additional file 3. Visual results of herbicides screening tests performed on some Italian Lolium spp. populations collected in wheat fields. [file 13007_2023_989_MOESM3_ESM.docx]
